# Flavivirus Cell Entry and Membrane Fusion

**DOI:** 10.3390/v3020160

**Published:** 2011-02-22

**Authors:** Jolanda M. Smit, Bastiaan Moesker, Izabela Rodenhuis-Zybert, Jan Wilschut

**Affiliations:** Department of Medical Microbiology, Molecular Virology Section, University Medical Center Groningen, University of Groningen, PO Box 30.001, 9700 RB Groningen, The Netherlands; E-Mails: b.j.s.moesker@med.umcg.nl (B.M.); i.a.rodenhuis-zybert@med.umcg.nl (I.R.-Z.); j.c.wilschut@med.umcg.nl (J.W.)

**Keywords:** flavivirus, membrane fusion, anionic lipids, negatively charged lipids, cell entry, receptor, dengue, West Nile virus

## Abstract

Flaviviruses, such as dengue virus and West Nile virus, are enveloped viruses that infect cells through receptor-mediated endocytosis and fusion from within acidic endosomes. The cell entry process of flaviviruses is mediated by the viral E glycoprotein. This short review will address recent advances in the understanding of flavivirus cell entry with specific emphasis on the recent study of Zaitseva and coworkers, indicating that anionic lipids might play a crucial role in the fusion process of dengue virus [1].

## 1. Introduction

Dengue virus (DENV) is a major emerging arthopod-borne pathogen causing a significant burden of disease in tropical and subtropical areas of the world [2,3]. DENV is a positive-sense RNA virus, belonging to the genus “Flaviviruses” of the family *Flaviviridae* [4]. The flavivirus genus comprises more than 70 viruses including, besides DENV, a number of other important human pathogens such as West Nile virus (WNV), tick-borne encephalitis virus (TBEV), Japanese encephalitis virus (JEV), and yellow fever virus (YFV). Flaviviruses are enveloped viruses, which enter their host cells through a process of receptor-mediated endocytosis and subsequent fusion from within the endosomal cell compartment. This fusion process is triggered by the mildly acidic pH within the lumen of the endosome [4]. In this short review, new insights in flavivirus cell entry and membrane fusion will be discussed. We will emphasize specifically the role of target membrane lipids in the membrane fusion activity of DENV [1].

## 2. Structure of Flaviviruses

Flaviviruses are small, icosahedral viruses. The viral genome consists of a single-stranded, positive-sense RNA molecule which is complexed to multiple copies of the capsid protein [5]. The nucleocapsid is surrounded by a host-derived lipid membrane, in which two transmembrane proteins are inserted, the major envelope glycoprotein E (53 kDa) and the membrane protein M (8 kDa). The M protein is a small proteolytic fragment of its precursor form prM (approximately 21 kDa) and is anchored into the viral membrane by two transmembrane helices [6,7]. In mature virions, the E glycoproteins are arranged in 90 homodimers with sets of three E head-to-tail homodimers that lie in 30 rafts and form a herringbone pattern [6]. The E ectodomain has three structurally distinct domains (DI, DII, DIII) that are connected by flexible hinge regions [8]. Neither E nor M interacts with the nucleocapsid in mature virions [9]. In infected cells, virions are initially assembled in an immature form with a distinct structural organization. In these particles, the E glycoprotein is associated with the glycoprotein prM and three of these heterodimers form one viral spike [6,10]. Virus particle maturation occurs during viral egress.

## 3. Flavivirus Cell Entry

### 3.1 Receptor Binding

The first step in the infectious cell entry pathway of flaviviruses involves binding of the E glycoprotein to a cellular receptor. Flaviviruses must recognize a ubiquitous cell surface molecule or utilize multiple receptors for cell entry as flavivirus infection has been observed in a variety of cell lines derived from different host species [11]. In recent years several attachment factors have been identified, indicating that flaviviruses may use multiple receptors for cell entry.

Negatively charged glycoaminoglycans, such as heparan sulfate, which are abundantly expressed on numerous cell types, are utilized as low-affinity attachment factors by several flaviviruses [12–18]. These interactions serve to concentrate the virus at the cell surface and are mediated by domain DIII of the E glycoprotein. Multiple other attachment factors have been identified for DENV in mammalian cells including heat-shock proteins 90 and 70 [19], neolactotetraosylceramide [20], CD14 [21], GRP78/BiP [22], 37-kDa/67-kDa laminin [23], and C-type lectins such as DC-SIGN (dendritic cell-specific intracellular adhesion molecule-3 (ICAM3)-grabbing non-integrin) [24–26], the mannose receptor [27], and C-type lectin domain family 5, member A (CLEC5, MDL-1) [28]. In mosquito cells, DENV has been shown to interact with heat-shock protein 70, R80, R67 and a 45-kDa protein [19,29,30]. Crystallographic studies on DENV-DC-SIGN complexes revealed that interaction with DC-SIGN is preferentially mediated through the carbohydrate moiety at Asn67 in EDII [31]. WNV has also been shown to interact with DC-SIGN and DC-SIGNR in dendritic cells [32,33]. Furthermore, WNV, JEV, and DENV, albeit to a lesser extent, have been documented to bind to α_v_β_3_ integrins expressed on mammalian cells, mediated through interaction with EDIII [34,35]. On the other hand, entry of WNV into embryonic mouse fibroblasts and hamster melanoma is independent of α_v_β_3_ integrin binding, suggesting that receptor molecule usage is strain-specific and/or cell type-dependent [36].

### 3.2 Entry of Flavivirus Particles into Cells

Flaviviruses enter cells through clathrin-mediated endocytosis. Single-particle tracking analysis of DENV particles in living cells revealed that DENV particles diffuse along the cell surface towards a pre-existing clathrin-coated pit [37]. This implies that virions roll over distinct attachment factors untill they bind to the entry receptor localized to clathrin hotspots at the cell surface or that the initially formed virus-receptor complex is transported towards a pre-existing clathrin-coated pit. Subsequently, the clathrin-coated pit evolves and the invagination in the plasma membrane is closed by membrane scission mediated by dynamin to form a clathrin-coated vesicle. The clathrin-coated vesicle is transported away from the plasma membrane after which the clathrin coat is released from the vesicle. Real-time microscopy analysis showed that DENV particles remain associated with clathrin for approximately 80 seconds [37]. Earlier evidence that flaviviruses utilize clathrin-mediated endocytosis for cell entry was obtained by ultrastructural studies showing the presence of Kunjin and YFV in coated pits [38,39]. Furthermore, inhibition of WNV infection was observed in cells treated with chemical inhibitors like chlorpromazine [40] that prevent clathrin-coated pit formation and in cells expressing dominant-negative mutants of Eps15, a protein which is involved in clathrin-coated pit formation [41,42]. The route of flavivirus cell entry appears to be dependent on the virus strain and the cell type as a recent report documented entry of DENV in mammalian cells independent of clathrin, caveolae and lipid rafts [43].

After clathrin-mediated uptake, the endocytic vesicle carrying the virus is delivered to early endosomes. Internalization of flavivirus particles occurs rapidly as a large fraction of WNV and DENV particles were observed to localize to early endosomes within five minutes post-entry [37,41]. Thereafter, the early endosome carrying the virus matures into a late endosome. For DENV, membrane fusion has been observed to occur primarily from within late endosomal compartments [37]. Membrane fusion was detected on average at 10–13 minutes after initiation of infection. It is important to note that the subcellular compartment from which membrane fusion occurs is most likely dependent on the pH-dependent membrane fusion properties of the virus and may therefore vary between individual DENV strains [37,44].

## 4. Molecular Mechanism of Membrane Fusion

### 4.1 Low-pH-induced Conformational Changes in the E Glycoprotein

The low-pH environment within endosomes triggers a series of molecular events within the E glycoprotein leading to membrane fusion of the viral membrane with the endosomal membrane and subsequent release of the nucleocapsid into the cell cytosol. Protonation of one or more histidine residues has been postulated to trigger the conformational changes of the E protein [45,46]. Indeed, studies on TBEV identified two conserved histidines (at position 146 and 323) that may act as pH sensors and destabilize the DI-DIII interface [47]. On the other hand, site-specific mutagenesis studies on WNV revealed that histidine residues are not required for the initiation of the conformational changes required for membrane fusion [48].

The conformational changes of the E glycoprotein that occur during the membrane fusion process of flaviviruses have been studied extensively [45,46,49]. The initial step in membrane fusion involves protonation-dependent disruption of the E protein rafts at the viral surface, including dissociation of E homodimers into monomers. This leads to the outward projection of DII and exposure of the fusion loop at the distal tip of DII to the target membrane. Subsequently, the E proteins insert their fusion loops into the outer leaflet of the membrane and three copies of E interact with one another via their fusion loops or DII domains to form an unstable trimer. The E trimers stabilize through additional interactions between the DI domains of the three E proteins [50]. Next, DIII is believed to fold back against the trimer to form a hairpin-like configuration. The energy released by these conformational changes induces the formation of a hemifusion intermediate, in which the monolayers of the interacting membranes are merged while the inner membranes are still intact. Finally, a fusion pore is formed and after enlargement of the pore, the nucleocapsid is released into the cytosol.

### 4.2 Role of Cholesterol in Flavivirus Membrane Fusion

Besides the mildly acidic endosomal pH, it is the composition of the target membrane that plays an important role in the membrane fusion process of flaviviruses. *In vitro* studies have revealed that flaviviruses such as TBEV and WNV have the capacity to fuse with artificial receptor-free lipid membranes (liposomes) consisting of phosphatidylcholine (PC) and phosphatidylethanolamine (PE) at low pH, albeit with low efficiency [51–53]. Addition of cholesterol to target membranes was found to have a strong promoting effect on the membrane fusion capacity of TBEV and WNV [51–54]. Subsequent virus-liposome coflotation studies have indicated that cholesterol stimulates the low-pH-triggered interaction of the E glycoprotein with lipid membranes [54,55]. The 3-hydroxyl group of cholesterol is important for this function [54]. Interestingly, and in contrast to alphaviruses, the E glycoprotein does not appear to directly interact with cholesterol in the target membrane [55]. These observations suggest that the promoting effect of cholesterol on membrane fusion is due to an overall change in the fluidity or physico-chemical properties of the target membrane. Although most studies were performed with TBEV and WNV, several recent studies show that DENV also has the capacity to interact and fuse with artifical membranes consisting of PC, PE, sphingolipids and cholesterol [56–58]. Cholesterol also plays an important role in facilitating efficient cell entry of flaviviruses as viral infectivity was found to be significantly impaired in cholesterol-depleted cells [36,59–61].

### 4.3 Role of Negatively Charged Lipids in Dengue Virus Membrane Fusion

A recent study from Zaitseva and coworkers showed that DENV may utilize anionic lipids as a cofactor during the low-pH-driven membrane fusion process of the virus [1]. The investigation, by these authors, of the lipid dependence of DENV membrane fusion was triggered by the observation that DENV does not fuse with the plasma membrane of mammalian cells under low-pH conditions whereas efficient fusion occurred with the plasma membrane of insect cells. A difference between these cell types is that insect cells have an unusually high concentration of anionic lipids in their plasma membrane. Accordingly, the authors found that addition of anionic lipids, such as bis(monoacylglycero)phosphate (BMP) and phosphatidylserine (PS), to the plasma membrane of mammalian cells facilitates low-pH-induced plasma membrane fusion of DENV. The role of anionic lipids in DENV membrane fusion was confirmed by *in vitro* virus-liposome fusion measurements, in which efficient membrane fusion was only observed using target membranes consisting of PC (70 mol%) and BMP or PS or phosphatidylglycerol (PG). Furthermore, anionic lipids were observed to act downstream of the formation of a restricted hemifusion intermediate and likely promote the opening of the fusion pore. Interestingly, in mammalian cells, anionic lipids are enriched in late endosomal compartments, thus this observation may also explain why DENV fusion is primarily initiated from within these compartments [37].

While the study of Zaitseva *et al.* is elegant and may explain a number of the characteristics of DENV cell entry, a word of caution seems justified. In earlier studies, negatively charged phospholipids have been observed to promote a number of viral membrane fusion reactions. First, PS has been suggested to serve as a receptor in cell entry of vesicular stomatitis virus [62], but more recent studies have challenged this view [63]. Furthermore, influenza virus was found to fuse quite efficiently with liposomes consisting of the anionic lipid cardiolipin in a low-pH-dependent manner [64]. However, subsequent detailed characterization of this fusion reaction revealed that it does not reflect the physiological fusion process of the virus. Specifically, the well-documented conformational change in the viral hemagglutinin (HA) turned out *not* to be involved [65]. Indeed, influenza virus pre-exposed to low pH in the absence of target membranes remained fusion-active with cardiolipin target liposomes [65,66], while such a low-pH pretreatment is known to result in a rapid and irreversible loss of fusion capacity—and infectivity—of the virus [66]. It thus appears that anionic lipids have a tendency to promote membrane fusion of enveloped viruses (and, for that matter, other biological membranes as well [67]), in a general, sometimes nonphysiological, manner [68]. The fact that, in the study of Zaitseva *et al.*, several structurally different negatively charged phospholipids stimulate fusion of DENV, including lipids—such as phosphatidylglycerol (PG)—that are not present in the natural endosomal target membrane of the virus does not seem to support the notion of a highly specific cofactor role of these lipids in the process [1]. On the other hand, the experiments of Zaitseva *et al*. are well-designed and convincing. In particular, the observation that the anionic lipids act at a stage downstream of the formation of the hemifusion intermediate [1] is reassuring, as this localizes the role of these lipids to the cytoplasmic half of the endosomal target membrane, which is the leaflet where negatively charged phospholipids are primarily located. Further studies will be required to elucidate whether indeed anionic phospholipids represent a necessary and sufficient factor involved in infectious DENV fusion from within acidic endosomes.

## 5. Particle Maturation Status and Infectivity

Flavivirus-infected cells are known to secrete a mixture of mature, immature and partially mature particles. The prM quantity appears to be different between flaviviruses; whereas high numbers of prM-containing particles have been described for WNV and DENV [53,69–75], low numbers were observed for TBEV [76]. A recent study showed that as much as 40% of all extracellular DENV particles derived from C6/36 mosquito cells are partially immature [77]. Overall, this demonstrates that flavivirus maturation during virus egress is rather inefficient. Upon assembly of immature virions in the ER, all virus particles are transported to the Golgi apparatus [78–82]. Within the mildly acidic environment of the Golgi, the virion undergoes a global conformational change leading to dissociation of E/prM heterodimers and formation of 90 homodimers [58,76,83]. This conformational change allows the host protease furin to cleave prM to M and a “pr” peptide [58,76,83,84]. The pr peptide dissociates from the particle upon release of the virion to the extracellular milieu [58,76,84,85]. It appears that furin processing of prM is rather inefficient and flavivirus particles that contain uncleaved prM proteins will, after the release of the virion to the extracellular milieu, reorganize back to form prM/E heterodimers at the viral surface.

Numerous functional studies have shown that fully immature particles are noninfectious [53,58,74–76,83,86]. However, we and others recently demonstrated that fully immature particles can be rendered infectious by antibodies [87,88]. We observed that the lack of infectivity of fully immature particles was found to be related to inefficient binding to the cell surface [88]. Upon cell entry, immature virions are efficiently processed by furin, the pr peptide presumably being released at the low-pH environment of endosomes [85]. Whereas proteolytic cleavage of prM is a prerequisite for viral infectivity, multiple studies have shown that complete cleavage is not required for infectivity [74,75,89,90]. In partially mature particles, the mature aspect of the virion is most likely responsible for virus cell binding and entry after which the processing of prM may occur within the target cell and membrane fusion may be initiated. The threshold for viral infectivity in relation to the number of prM proteins present at the viral surface is as yet not understood.

## 6. Concluding Remarks

In recent years, our knowledge on the cell entry properties and membrane fusion activity of flaviviruses has improved significantly. Microscopic analysis of virus-infected cells has taught us how viruses hijack cellular pathways to deposit their genome into the host cell cytosol. Also, biochemical and structural approaches have identified important steps in the membrane fusion process of flaviviruses. The observation that viral infectivity is dependent on the maturation status of the particle is interesting and adds yet another layer of complexity to flavivirus cell entry. Future studies should be directed at a further dissection of the dynamics of flavivirus entry into human target cells and a detailed understanding of, not only the conformational changes of the E glycoprotein during membrane fusion, but also the role of target membrane lipids in the fusion process. Furthermore, given the substantial levels of prM in wt DENV, it will be crucial to gain more insight in the role of immature or partially immature virions in viral infectivity. A detailed understanding of the cell entry and membrane fusion process of flaviviruses is important and will facilitate the generation of novel antiviral drugs that prevent early steps of infection.
